# Head-to-Head Comparison of Modular Vaccines Developed Using Different Capsid Virus-Like Particle Backbones and Antigen Conjugation Systems

**DOI:** 10.3390/vaccines9060539

**Published:** 2021-05-21

**Authors:** Laurits Fredsgaard, Louise Goksøyr, Susan Thrane, Kara-Lee Aves, Thor G. Theander, Adam F. Sander

**Affiliations:** 1 Centre for Medical Parasitology, Department of Immunology and Microbiology, Faculty of Health and Medical Sciences, University of Copenhagen, 2200 Copenhagen, Denmark; laurits@fredsgaardlarsen.com (L.F.); louiseg@sund.ku.dk (L.G.); kara-lee@sund.ku.dk (K.-L.A.); thor@sund.ku.dk (T.G.T.); 2 AdaptVac Aps, 2970 Hørsholm, Denmark; susanthrane@gmail.com

**Keywords:** vaccine, virus-like particle, conjugation system, vaccine design, modular vaccine platform

## Abstract

Capsid virus-like particles (cVLPs) are used as molecular scaffolds to increase the immunogenicity of displayed antigens. Modular platforms have been developed whereby antigens are attached to the surface of pre-assembled cVLPs. However, it remains unknown to what extent the employed cVLP backbone and conjugation system may influence the immune response elicited against the displayed antigen. Here, we performed a head-to-head comparison of antigen-specific IgG responses elicited by modular cVLP-vaccines differing by their employed cVLP backbone or conjugation system, respectively. Covalent antigen conjugation (i.e., employing the SpyTag/SpyCatcher system) resulted in significantly higher antigen-specific IgG titers compared to when using affinity-based conjugation (i.e., using biotin/streptavidin). The cVLP backbone also influenced the antigen-specific IgG response. Specifically, vaccines based on the bacteriophage AP205 cVLP elicited significantly higher antigen-specific IgG compared to corresponding vaccines using the human papillomavirus major capsid protein (HPV L1) cVLP. In addition, the AP205 cVLP platform mediated induction of antigen-specific IgG with a different subclass profile (i.e., higher IgG2a and IgG2b) compared to HPV L1 cVLP. These results demonstrate that the cVLP backbone and conjugation system can individually affect the IgG response elicited against a displayed antigen. These data will aid the understanding and process of tailoring modular cVLP vaccines to achieve improved immune responses.

## 1. Introduction

Life-long protective antibody responses have generally only been achieved by live-attenuated vaccines [1] whose immunogenicity to a large extent has been ascribed to the infectious nature of the inoculum [2]. However, immunological data generated by the licensed human papillomavirus (HPV) vaccines could indicate that structural properties of the native virion may also constitute an essential factor for the activation of the immune system [2,3,4]. The HPV vaccines are built from multiple copies of the major capsid protein (L1), which during recombinant expression self-assembles into capsid virus-like particles (cVLPs) [5,6,7]. HPV L1 cVLPs are highly immunogenic and can induce potent anti-L1 antibody responses lasting for decades, even after a single immunization [2,8,9,10,11]. The structural similarity of cVLPs to native viruses has been established as a reason for their high immunogenicity. In particular, their size (20–200 nm diameter) allow for direct draining to the lymph nodes, and the highly repetitive surface geometry enables efficient B cell receptor crosslinking [3,4,12,13,14,15,16,17,18]. Thus, multiple studies suggest that multivalency and the repetitiveness of an antigen are key to induce potent and long-lived antibody responses [12,19]. Consequently, several strategies have been pursued to exploit cVLPs as scaffolds for presentation of heterologous antigens, and modular vaccine platforms have emerged by which the vaccine antigen is conjugated by different methods to pre-assembled cVLPs [20,21].

**Figure 1:**
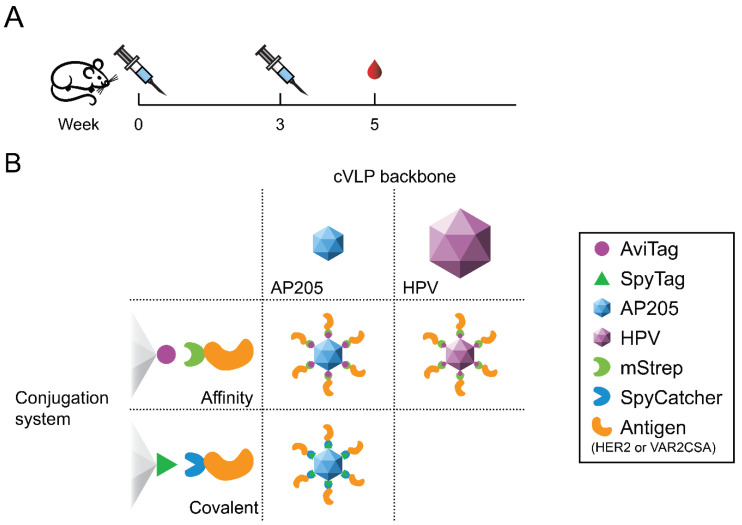
Study setup. (**A**) Groups of mice (*n* = 6) were immunized prime-boost and serum was collected 2 weeks after the final immunization to evaluate the vaccine-induced IgG response elicited against the cVLP-displayed antigen. (**B**) Capsid virus-like particles (cVLPs) displaying either SpyTag or biotinylated AviTag were mixed with antigens genetically fused to SpyCatcher or monomeric streptavidin (mStrep), respectively. Two different model antigens (HER2 and VAR2CSA) were used. The AP205 cVLP consists of 180 subunits and has a diameter of approximately 30 nm, while the human papillomavirus (HPV) major capsid protein (L1) cVLP consists of 360 subunits and has a diameter of approximately 55 nm.

To this end, we previously developed HPV L1 cVLPs displaying one biotinylated biotin acceptor site (AviTag™) per subunit, which enabled affinity-based conjugation of antigens genetically fused to monomeric streptavidin (mStrep) [22,23,24]. This modular vaccine platform significantly increased the immunogenicity of the malaria antigen, VAR2CSA [25]. More recently, ‘split-protein’-based conjugation systems have been developed [26,27,28,29,30,31,32,33,34,35]. These systems make use of specific peptide (Tag) and protein (Catcher) binding partners, which upon mixture react spontaneously to form an irreversible isopeptide bond [26,28,33,36]. The most widely used split-protein system (i.e., SpyTag/SpyCatcher) has been used to develop several cVLP-based vaccines [37,38,39]. Specifically, the AP205 cVLP has been engineered to display a SpyTag on the surface of each subunit, allowing subsequent conjugation of SpyCatcher-fused antigens [40,41,42,43]. While both the biotin/mStrep and the SpyTag/SpyCatcher system have the advantage of enabling unidirectional antigen display of large and complex protein antigens, the covalent conjugation method (i.e., SpyTag/SpyCatcher) seems preferable because of the more stable antigen binding, although this assumption has not been experimentally validated. To investigate this, we exchanged the 13 amino acid (aa) SpyTag sequence at the N-terminus of the AP205 coat protein with the 15 aa AviTag™ biotin acceptor sequence to create AviTag-AP205. Hereafter, the AviTag-AP205 and AviTag-HPV L1 platforms were used to deliver the same antigen conjugated either by a covalent bond or by the high-affinity biotin/mStrep interaction. Thus, a controlled head-to-head comparison of modular vaccines differing only in their employed conjugation system (covalent vs. affinity-based) or cVLP backbone (AP205 vs. HPV) was performed (Figure 1). The comparative assessment was based on analysis of IgG responses elicited against two cVLP-displayed model antigens, the *Plasmodium falciparum* VAR2CSA [44] and the human epidermal growth factor receptor 2 (HER2) [45].

## 2. Materials and Methods

### 2.1. Design, Expression and Purification of cVLPs

SpyTag-AP205 cVLPs were expressed and purified as previously described [42]. AviTag-AP205 was constructed by genetic fusion of a biotin acceptor sequence (AviTag™ (Avidity, Aurora, CO, USA), GLNDIFEAQKIEWHE) to the N-terminal end of the *Acinetobacter phage* AP205 coat protein (GenBank ID: NP_085472.1) with a flexible linker in between (GSGTAGGGSGS). The AviTag-AP205 expression sequence was cloned into a modified pET-15b vector and transformed into One Shot^®^ BL21 Star™ (DE3) (New England Biolabs, Ipswich, MA, USA) cells. Both SpyTag-AP205 and AviTag-AP205 cVLPs were expressed and purified by ultracentrifugation as previously described [42]. cVLP-containing fractions were pooled and dialyzed in 1xPBS, pH 7.4 using Spectra/Por™ Cellulose Ester 1000 kDa MWCO dialysis tubing (Spectrum Chemical, New Brunswick, NJ, USA). Protein concentration was determined with Pierce™ BCA Protein Assay Kit (Thermo Fisher Scientific, Waltham, MA, USA).

HPV16 L1 cVLPs containing an AviTag™ (Avidity, Aurora, CO, USA) in the FG-loop [46] was previously described [25]. The pAcGP67A/AviTag-AP205-HPV transfer plasmid was co-transfected with flashBAC GOLD™ (Oxford Expression Technologies Ltd., Oxford, UK) into Sf9 cells using Lipofectamine™ 2000 (Invitrogen, Carlsbad, CA, USA). Recombinant Baculovirus harvested from the supernatant was used to generate a high-titer virus stock for infection of High Five™ cells (Thermo Fischer Scientific, Waltham, MA, USA). The infected High Five™ cells were incubated for 48 h at 28 °C, 130 rpm in Insect-XPRESS™ medium (Lonza, Basel, Switzerland). Cells were harvested by centrifugation (9000× *g*) and resuspended in Dulbecco’s PBS with calcium and magnesium (Sigma-Aldrich, St. Louis, MO, USA) containing cOmplete™ Mini EDTA-free Protease Inhibitor Cocktail (F. Hoffmann-La Roche, Basel, Switzerland). For cVLP maturation, 0.5% Triton X-100, 0.1% Benzonase, 25 mM ammonium sulphate pH 9 and 4 mM MgCl was added to the suspension, followed by incubation for 18 h at 37 °C with rotation. The cell lysate was chilled on ice and adjusted to 0.8 M NaCl, incubated on ice for 10 min and centrifuged at 5000× *g* for 5 min at 4 °C [47]. cVLPs were purified by ultracentrifugation through Optiprep™ (Sigma-Aldrich, St. Louis, MO, USA) density gradient as previously described [25]. AviTag-AP205 and AviTag-HPV were incubated at 30 °C with biotin (Avidity, Aurora, CO, USA) and biotin ligase (BirA, Avidity, Aurora, CO, USA) according to manufacturer’s instructions. Excess biotin was removed by dialysis into PBS (0.32 M NaCl, 0.02% Polysorbate 80, pH 7.4).

### 2.2. Antigen Protein Expression and Purification

The design, expression and purification of SpyCatcher-HER2 has previously been described [45]. Additionally, the SpyCatcher-HER2 was further purified by size exclusion chromatography (SEC; 1xPBS, HiLoad 26/600 Superdex 200 pg, GE Healthcare, Chicago, IL, USA) to obtain a monomeric fraction of the protein. mStrep-HER2 was constructed by genetic fusion of mStrep (GenBank ID: 4JNJ_A) to the N-terminus of the extracellular domain of HER2 (aa23-652, GenBank ID: NP_004439) separated by a flexible linker (GGS). A hexa-histidine purification tag was added to the C-terminus. The mStrep-HER2 gene sequence was cloned into pAcGP67A transfer vector (BD Biosciences, San Jose, CA, USA). The pAcGP67A/mStrep-HER2 transfer plasmid was co-transfected with flashBAC GOLD™ (Oxford Expression Technologies Ltd., Oxford, UK) into Sf9 cells using Lipofectamine™ 2000 (Invitrogen, Carlsbad, CA, USA). Recombinant Baculovirus harvested from the supernatant was used to generate a high-titer virus stock for infection of High Five™ cells (Thermo Fischer Scientific, Waltham, MA, USA). Infected High Five™ cells were incubated for 28 h at 28 °C, 130 rpm in Insect-XPRESS™ medium (Lonza, Basel, Switzerland). The recombinant protein was harvested from the supernatant by centrifugation at 10,000× *g* for 10 min at 4 °C, concentrated and buffer exchanged (1xPBS supplemented with 60 mM Imidazole) using a QuixStand™ benchtop system (10,000 MWCO hollow fiber cartridge, surface area 650 cm^2^, GE healthcare, Chicago, IL, USA). The recombinant protein was purified by immobilized metal affinity chromatography (IMAC; step-elution: 1xPBS supplemented with 60 mM Imidazole for binding and 600 mM imidazole for elution, HisTrap™ HP 5 mL, GE Healthcare, Chicago, IL, USA) and SEC (1xPBS, HiLoad 26/600 Superdex 200 pg, GE Healthcare, Chicago, IL, USA) to obtain a monomeric fraction of the protein.

mStrep-VAR2CSA was constructed by fusing mStrep (GenBank ID: 4JNJ_A) to the N-terminus of the ID1-ID2a domains of VAR2CSA (FCR3 strain, GenBank ID: GU249598), separated by a flexible linker (GGS). A hexa-histidine purification tag was added to the C-termini [25]. The gene expressing sequence was cloned into a modified pET-15b vector and transformed into One Shot^®^ BL21 Star™ (DE3) (New England Biolabs, Ipswich, MA, USA) cells. mStrep-VAR2CSA was expressed in 2xYT media containing 100 µg/mL ampicillin. Cells were grown at 37 °C until log-phase (OD_600_ ≈ 0.6), followed by induction with Isopropyl β-D-1-thiogalactopyranoside at a final concentration of 0.1 mM and incubated overnight at 20 °C with shaking. Cells were harvested by centrifugation at 10,000× *g* for 10 min at 4 °C, resuspended in 1xPBS (supplemented with 60 mM Imidazole and 0.4 M NaCl) and lysed by sonication. The lysate was clarified by centrifugation (40,000× *g* for 30 min at 4 °C) and purified by IMAC as described above. mStrep-VAR2CSA was further purified by ion exchange chromatography (0–1 M NaCl gradient; HiTrap SP HP, GE Healthcare, Chicago, IL, USA).

### 2.3. Formulation and Purification of the cVLP Vaccines

cVLPs were mixed with a 2× molar excess of antigen, then incubated overnight at 4 °C with gentle shaking. LPS was removed from the vaccines by phase extraction using Triton X-114 as previously described [48]. Unbound antigen was removed by density gradient ultracentrifugation (OptiPrep™, Sigma-Aldrich, St. Louis, MO, USA). Fractions containing the vaccine was dialyzed using a Spectra/Por™ Cellulose Ester 1000 kDa MWCO dialysis tubing (Spectrum Chemical, New Brunswick, NJ, USA) in PBS or PBS supplemented with 0.4 M NaCl for HER2 and VAR2CSA vaccines respectively. cVLP-bound antigen concentration as well as the coupling efficiency was determined by SDS-PAGE densitometric analysis. For DLS analysis (DynaPro NanoStar, Wyatt Technology, Santa Barbara, CA, USA), the sample was spun at 16,000× g for 2 min, then loaded into a disposable cuvette. Samples were run with 20 acquisitions of 7 s each. The estimated diameter of the cVLP vaccine population and the percent polydispersity (%Pd) was calculated with Wyatt DYNAMICS software (v7.10.0.21, Wyatt Technology, Santa Barbara, CA, USA).

### 2.4. Animal Immunizations

Experiments were authorized by the National Animal Experiments Inspectorate (Dyreforsøgstilsynet, license no. 2018-15-0201-01541) and performed according to national guidelines. Groups of 6 female FVB/NRj mice, 6–8 weeks old, were obtained from Janvier Labs and housed in a specific pathogen-free facility. Animals were acclimatized at the facility for at least 1 week prior to their use. Mice were immunized intramuscularly in a prime-boost regimen, with 3 weeks in between. No extrinsic adjuvants were used. Immunizations were normalized to contain comparable amounts of cVLP-bound antigen by excluding mass contributed by SpyCatcher/mStrep. The mice were immunized with either 2 µg or 1.19 µg cVLP-bound HER2 or VAR2CSA, respectively. Blood samples were collected 2 weeks after the boost immunization. Sera was obtained by spinning the blood twice at 800× g for 8 min at 8 °C.

### 2.5. IgG Response Measured by ELISA

96-well Nunc Maxisorp™ flat-bottom plates (Invitrogen, Carlsbad, CA, USA) were coated overnight at 4 °C with either recombinant HER2 (aa23-652, GenBank ID: NP_004439) or VAR2CSA (ID1-ID2a, FCR3 strain, GenBank ID: GU249598). HER2 coat protein was produced like its Catcher-counterpart [45], while VAR2CSA is described elsewhere [49]. Plates were blocked for one hour in 0.5% skim milk powder in PBS at room temperature (RT). Sera were diluted 100× and added to the well in threefold dilutions in blocking buffer and incubated for 1 h at RT. Plates were washed with 1xPBS three times in between steps. Plates were incubated for 1 h at RT with HRP-conjugated secondary antibodies targeting either total mouse IgG, IgG1, IgG2a or IgG2b (Invitrogen, Carlsbad, CA, USA). Plates were developed with TMB X-tra substrate (Kem-En-Tec, Taastrup, Denmark). OD_450_ was measured with a HiPo MPP-96 microplate photometer (Biosan, Riga, Latvia).

### 2.6. Statistical Analysis

Area under the curve (AUC) was determined with the log_10_ transformed dilution factor using the Python library SciPy’s trapezoid rule function. Statistical significance was determined by two-tailed Mann-Whitney U test. A difference with a *p*-value ≤ 0.05 was considered significant.

## 3. Results

### 3.1. The Vaccine-Induced IgG Profile Is Affected by the cVLP Backbone

**Figure 2:**
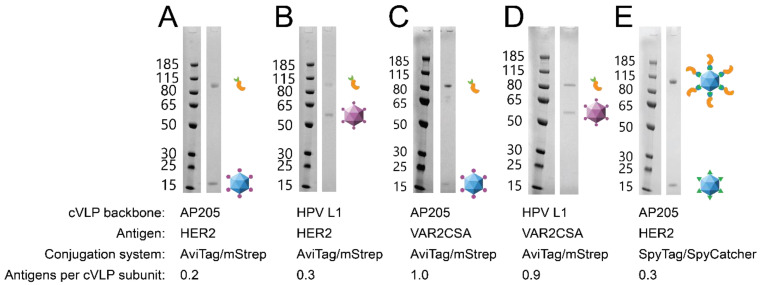
Vaccine coupling efficiency. After purification by ultracentrifugation and dialysis, the coupling efficiency of each vaccine was measured by SDS-PAGE densitometry. During reducing SDS-PAGE, the affinity-based AviTag:mStrep-conjugation is disassembled (**A**–**D**), while the covalent SpyTag:SpyCatcher-conjugation is maintained (**E**). (**A**) AviTag-AP205:mStrep-HER2. (**B**) AviTag-HPV:mStrep-HER2. (**C**) AviTag-AP205:mStrep-VAR2CSA. (**D**) AviTag-HPV:mStrep-VAR2CSA. (**E**) SpyTag-AP205:SpyCatcher-HER2. The number of antigens per cVLP subunit was calculated based on the relative intensities of protein bands present in each lane (see Supplementary Table S1 for a detailed explanation).

**Figure 3:**
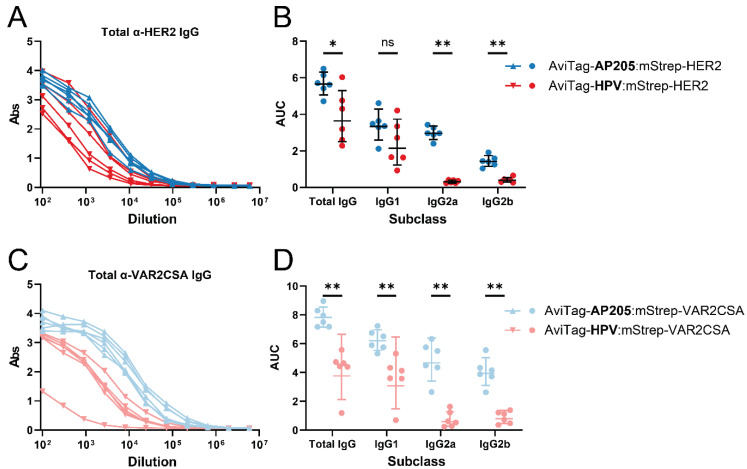
Effect of the cVLP backbone on IgG profile. Mice (*n* = 6) were immunized with antigen coupled to either HPV or AP205 through the non-covalent AviTag:mStrep interaction. The doses of HER2 and VAR2CSA were 2 µg and 1.19 µg antigen respectively. Antigen specific antibodies were quantified by ELISA. (**A**) Total anti-HER2 IgG dilution curves and (**B**) Area under the curve (AUC) for Total IgG, IgG1, IgG2a and IgG2b. (**C**) Total anti-VAR2CSA IgG dilution curves and (**D**) AUC for total IgG, IgG1, IgG2a and IgG2b. Each dot represents one animal. Full dilution curves are available in Supplementary Figure S2. Not significant (ns) *p* > 0.5; * *p* ≤ 0.5; ** *p* < 0.01.

Both HER2 and VAR2CSA were coupled (using the AviTag:mStrep system) to HPV and AP205 cVLP backbones, respectively, to investigate whether the cVLP backbone could influence the IgG response elicited against the displayed antigen. To ensure that the head-to-head compared vaccines were comparable in terms of their antigen display density, the coupling efficiency (antigens bound per cVLP subunit) was determined by densitometric analysis of SDS-PAGE. Specifically, the number of antigens bound per cVLP subunit was calculated from the theoretical molecular mass of the proteins and their relative quantity in the purified vaccine (see Supplementary Table S1 for a detailed explanation). This analysis revealed that the pairwise compared vaccines had similar purity and antigen coupling efficiency (HER2-vaccines: 0.2–0.3 antigens per cVLP subunit; VAR2CSA-vaccines: 0.9–1.0 antigens per cVLP subunit) (Figure 2). Moreover, a centrifugation test (2 min at 16,000× *g*), known empirically to pellet larger antigen:cVLP aggregates, was used to assess the aggregation state of the vaccines. This test did not show any measurable aggregation of any of the vaccines (data not shown). Finally, dynamic light-scattering (DLS) analysis showed that the antigen:cVLP complexes formed predominantly monodisperse particles (Supplementary Figure S1). Groups of mice (n = 6) were immunized prime-boost and the serum was analyzed for antigen-specific IgG by ELISA. For both HER2 and VAR2CSA, the vaccines based on the AP205 cVLP backbones induced higher antigen-specific IgG levels compared to the corresponding vaccines based on the HPV cVLP backbone (Figure 3A,C). Further analyses revealed a pronounced difference in the subclass profile of the antigen-specific IgG induced by the AP205 and HPV cVLP based vaccines, respectively. In particular, whereas the VAR2CSA and HER2 vaccines based on AP205 cVLPs induced high levels of antigen-specific IgG1, IgG2a and IgG2b, corresponding vaccines based on HPV cVLPs failed to elicit IgG2a and IgG2b (Figure 3B,D).

### 3.2. Covalent Coupling Yields a Higher Antigen-Specific IgG Titer

**Figure 4:**
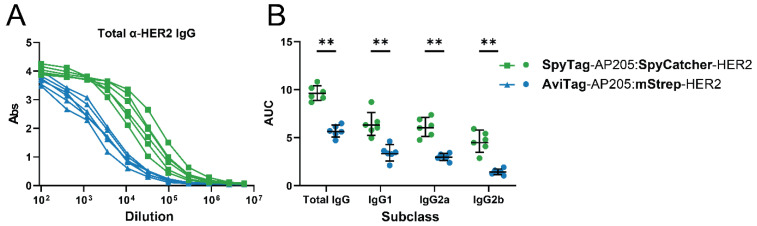
Effect of conjugation system on IgG profile. Mice (*n* = 6) were immunized with 2 µg HER2 coupled to AP205 cVLPs using either a covalent (SpyTag/SpyCatcher) or affinity-based (AviTag/mStrep) conjugation system. HER2-specific antibodies were quantified by ELISA. (**A**) Total IgG dilution curves and (**B**) AUC for total IgG, IgG1, IgG2a and IgG2b. Each dot represents one animal. Full dilution curves are available in Supplementary Figure S2. ** *p* < 0.01.

We next compared induction of IgG by two HER2 vaccines attached to AP205 by either the SpyTag/SpyCatcher system forming a covalent bond between the particle and the antigen complex or the AviTag system based on a high affinity interaction. On average the SpyTag-AP205:SpyCatcher-HER2 and AviTag-AP205:mStrep-HER2 vaccines displayed 54 and 43 HER2 antigens per cVLP, respectively (Figure 2A,E). The vaccine employing the SpyTag/SpyCatcher system induced significantly higher levels of anti-HER2 IgG than the vaccine where HER2 was coupled to the particle through the AviTag system (Figure 4A). The difference could be ascribed to a higher induction of IgG1, IgG2a and IgG2b (Figure 4B).

## 4. Discussion

The emergence of modular cVLP-based vaccine platforms creates a need for comparative studies investigating the immunological influence of using specific cVLP backbones and conjugation systems. cVLPs differ in multiple ways which could potentially affect their intrinsic immunogenicity as well as their capacity for antigen display. AP205 and HPV L1 cVLPs are produced in different expression systems (*Escherichia coli* vs. *Trichoplusia ni*) and differ in size/valency (30 nm/180 subunits vs. 55 nm/360 subunits, respectively). Previous studies have shown that optimal immune responses are induced against repetitive epitopes (at least 12–16) spaced by 5–10 nm [50,51]. It is thus possible that the increased size and valency of the HPV L1 cVLP could confer an advantage over the smaller AP205 cVLP. Furthermore, different repertoires of T cell epitopes contained in the cVLP backbone may cause varying activation of CD4+ T cells, which can help to enhance the immune response (including the antibody response) to the displayed antigen. In this study, head-to-head comparison of the HPV L1 and AP205 cVLP platform showed significantly higher antigen-specific antibody responses when using the AP205 cVLP platform for antigen display. Additionally, this platform promoted the induction of a Th1-type immune response towards the displayed antigen as evidenced by induction of IgG2a and IgG2b. Bacteriophage derived cVLPs like AP205, are known to encapsulate host cell RNA during their recombinant expression. This bacterial RNA acts as an intrinsic adjuvant, activating toll-like receptor 7 and 8, promoting Th1-type responses [48,52,53]. In immunization studies, induction of IgG2a and IgG2b has been correlated with increased protection against both infectious diseases and cancer [53,54,55,56,57,58]. Thus, the selection of a particular cVLP backbone might be key in achieving the desired type of immune response, and in this regard *E. coli* produced cVLPs may hold a specific advantage over cVLPs produced in eukaryotic expression platforms.

Regarding the two conjugation systems compared in this study (i.e., covalent vs. affinity-based), our analyses indicated an equal ability for achieving high-density and unidirectional display of the large HER2 protein. Nonetheless, immunization studies showed that the HER2-cVLP vaccine using the split-protein conjugation system (covalent conjugation) elicited significantly higher antigen-specific antibody responses compared to the HER2-cVLP vaccine developed using affinity-based conjugation (AviTag:mStrep). A likely explanation could be that the affinity-interaction is not stable in vivo, whereby the HER2 antigen is not presented in a high-density, multivalent cVLP format. In fact, the affinity of mStrep to biotin (K_d_~0.85 nM) is reduced compared to native Streptavidin (K_d_ ~ 10 fM) [59]. An alternative explanation could be that circulating biotin compete with the interaction with the mStrep-HER2 antigen. Another disadvantage of the AviTag:mStrep system is the scalability, which is limited by the need for biotinylation of the cVLP prior to antigen coupling.

This head-to-head study demonstrates how the choice of cVLP backbone and conjugation system can both significantly affect the antibody response elicited against a displayed antigen. In this study, vaccines based on AP205 cVLPs elicited stronger and more balanced IgG responses than vaccines employing the HPV cVLP backbone. This difference likely results from the fact that bacterial RNA is encapsidated in the lumen of AP205 cVLPs during their production in *E. coli*. It remains to be investigated if the AP205 cVLP platform would also induce higher levels of antibodies (including other isotypes) upon administration via different immunization routes and whether the two cVLP platforms differ in their ability for activating cellular immune responses.

## Data Availability

Data is contained within the article or supplementary material.

## Supplementary Materials

The following are available online at https://www.mdpi.com/article/10.3390/vaccines9060539/s1, Supplementary Figure S1: Dynamic light scattering of mStrep-HER2 vaccines. Supplementary Figure S2: Full dilution curves of antibody response with respect to IgG subclasses. Supplementary Table S1: Detailed explanation on how the coupling efficiency was calculated.
