# Supplementary material for: Head-to-Head Comparison of Modular Vaccines Developed Using Different Capsid Virus-Like Particle Backbones and Antigen Conjugation Systems
Source: Vaccines (Basel). 2021 May 21;9(6):539. doi: 10.3390/vaccines9060539 (PMC8224050; doi:10.3390/vaccines9060539)
Supplement: Supplementary file 1 [file vaccines-09-00539-s001.zip › vaccines-1205487-supplementary.pdf]

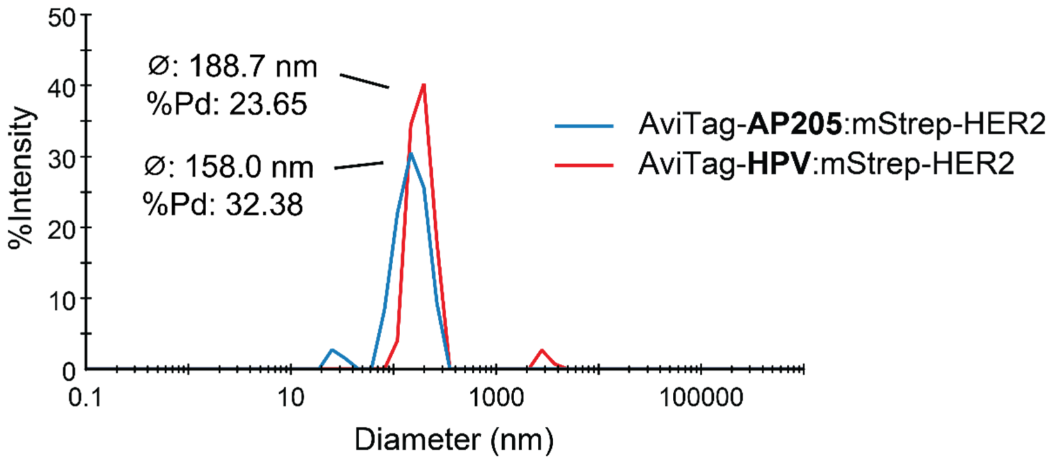

Figure S1. Dynamic light scattering of mStrep-HER2 vaccines: Regularization graph shows the size distributions of the mStrep-HER2 vaccines (AviTag-AP205:mStrep-HER2 (blue) and AviTag-HPV:mStrep-HER2 (red)).

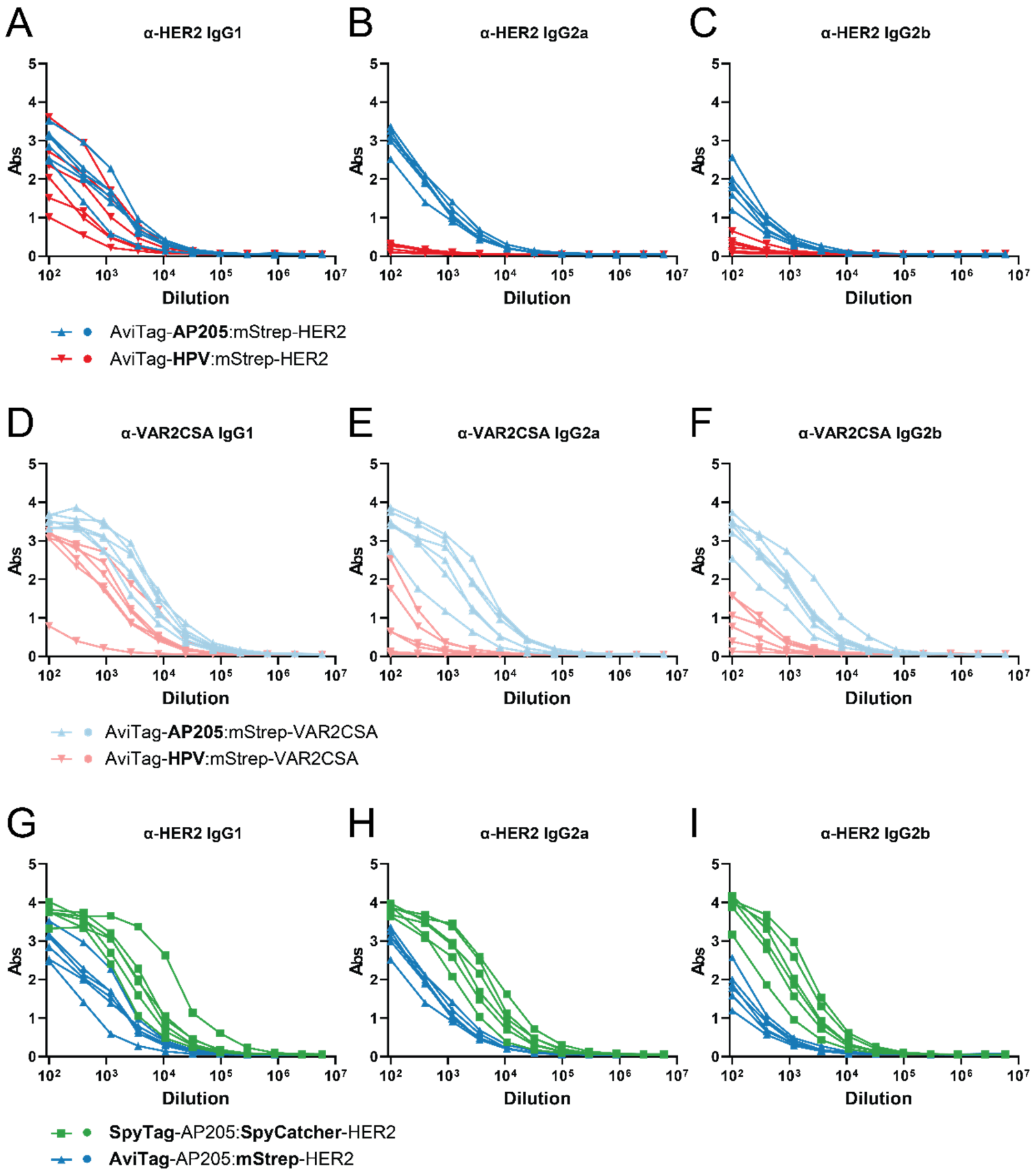

A
α-HER2 IgG1
B
α-HER2 IgG2a
C
α-HER2 IgG2b
Abs
Dilution
AviTag-AP205:mStrep-HER2
AviTag-HPV:mStrep-HER2
D
α-VAR2CSA IgG1
E
α-VAR2CSA IgG2a
F
α-VAR2CSA IgG2b
AviTag-AP205:mStrep-VAR2CSA
AviTag-HPV:mStrep-VAR2CSA
G
α-HER2 IgG1
H
α-HER2 IgG2a
I
α-HER2 IgG2b
SpyTag-AP205:SpyCatcher-HER2
AviTag-AP205:mStrep-HER2

Figure S2. Effect of cVLP backbone and conjugation system on IgG profile. Full dilution curves showing the antigen specific antibody response with respect to different IgG sub-classes.

Table S1. Detailed explanation on how the coupling efficiency was calculated.

**Vaccine**	**Protein Mass (kDa)**		**Relative Protein Quantity**		**Coupling Efficiency**
	**AviTag-cVLP**	**mStrep-Antigen**	**AviTag-cVLP**	**mStrep-Antigen**	
AviTag-AP205:mStrep-HER2	16.5	84	1	1.23	0.2
AviTag-HPV:mStrep-HER2	56	84	1	0.45	0.3
AviTag-AP205:mStrep-VAR2CSA	16.5	85.4	1	5.48	1.0
AviTag-HPV:mStrep-VAR2CSA	56	85.4	1	1.37	0.9

**Vaccine**	**Protein Mass (kDa)**			**Relative Protein Quantity**		**Coupling Efficiency**
	**SpyTag-cVLP**	**SpyCatcher-Antigen**	**SpyTag-cVLP :SpyCatcher-Antigen**	**SpyTag-cVLP**	**SpyTag-cVLP :SpyCatcher-Antigen**	
SpyTag-AP205:SpyCatcher-HER2	16.5	83	99.5	1	2.54	0.3

The coupling efficiency (number of antigens bound per cVLP subunit) was determined by SDS-PAGE densitometric analysis. Specifically, Image Lab 6.0.1 (BioRad) was used to measure the quantity of protein bands in each lane of the SDS-PAGE gel. A disk size of 8.0 mm was used for background subtraction. For the vaccine utilizing the SpyTag/SpyCatcher system, the conjugated antigen does not dissociate from the cVLP subunit during reduced SDS-PAGE. On the contrary, for vaccines utilizing the AviTag/mStrep system, the antigen will dissociate during reduced SDS-PAGE. For this reason, the exact method of calculating the coupling efficiency differs slightly between the two systems, as seen below.

$$\text{Antigen/cVLP ratio}_{\text{AviTag-HPV:mStrep-HER2}} = \frac{\frac{0.45}{84\ \text{kDa}}}{\frac{1}{56\ \text{kDa}}} = 0.3$$

$$\text{Antigen/cVLP ratio}_{\text{SpyTag-AP205:SpyCatcher-HER2}} = \frac{\frac{2.54}{99.5\ \text{kDa}}}{\frac{2.54}{99.5\ \text{kDa}} + \frac{1}{16.5\ \text{kDa}}} = 0.3$$
